# Cross-country differences in the association between diabetes and disability

**DOI:** 10.1186/2251-6581-13-3

**Published:** 2014-01-06

**Authors:** Shervin Assari, Reza Moghani Lankarani, Maryam Moghani Lankarani

**Affiliations:** 1 Department of Health Behavior and Health Education, University of Michigan School of Public Health, 1415 Washington Heights, Ann Arbor, MI 48109-2029, USA; 2 Center for Research on Ethnicity, Culture and Health, School of Public Health, University of Michigan, 1415 Washington Heights, Ann Arbor, MI 48109-2029, USA; 3 Tehran University of Medical Sciences, Tehran, Iran; 4 Medicine and Health Promotion Institute, Tehran, Iran; 5 Universal Network for Health Information Dissemination and Exchange (UNHIDE), Tehran, Iran

**Keywords:** Diabetes, Socio-economics, Disability, Exercise, Obesity, Cross country study

## Abstract

**Purpose:**

This study tested possible cross-country differences in the associations between diabetes and activities of daily living (ADLs), and possible confounding / mediating effects of socio-economic status, obesity, and exercise.

**Methods:**

Data came from Research on Early Life and Aging Trends and Effects (RELATE). The study included a total number of 25,372 community sample of adults who were 40 years or older. We used data from community based surveys in seven countries including China, Mexico, Barbados, Brazil, Chile, Cuba, and Uruguay. Demographics (age and gender), socio-economic status (education and income), obesity, exercise, and ADL (bath, dress, toilet, transfer, heavy, shopping, meals) were measured. Self-reported data on physician diagnosis of diabetes was the independent variable. We tested if diabetes is associated with ADL, before and after adjusting for socio-economics, obesity, and exercise in each country.

**Results:**

Based on *Model I* (age and gender adjusted model), diabetes was associated with limitation in at least one ADL in Mexico, Barbados, Brazil, Chile, Cuba, and Uruguay, but not China. Based on *Model II* that also controlled for education and income, education explained the association between diabetes and limitation in ADL in Mexico and Uruguay. Based on *Model III* that also controlled for exercise and obesity, in Cuba and Brazil, exercise explained the link between diabetes and limitation in performing ADLs. Thus, the link between diabetes and ADL was independent of our covariates only in Chile and Barbados.

**Conclusions:**

There are cross-country differences in the link between diabetes and limitation in ADL. There are also cross-country differences in how socio-economic status, obesity, and exercise explain the above association.

## Introduction

About three hundred fifty million people have diabetes worldwide [1]. With a direct medical cost of 465 billion U.S. dollars in the year 2011, diabetes is responsible for more than 10% of total healthcare expenditures in adults [2].

People with diabetes are more likely to experience limitations in activities of daily living (ADL), mobility, and role functioning [3-5]. Given the growing rate of diabetes and its associated disability burden in the world [2], for different reasons, more research is still needed on the link between diabetes and ADLs [6].

First, research on disability among people with diabetes may suggest avenues for reducing the disability attributable to this common chronic disease [3]. Second, although diabetes has been consistently related to a broad spectrum of health outcomes, including quality of life, ADL and mobility disability [7], and lower extremity function [8], it is not clear if the association between diabetes and ADL remains significant after controlling for other factors, and also it is not clear if the same link exits in different countries [9]. Third, a large proportion of a major part of evidence on the link between diabetes and burden of mobility-related disability has originated from studies conducted on patients, or disabled individuals [9]. However, clinical samples and community individuals vary on many factors and there is a need to show the ADLs among individuals with diabetes in a community sample. Finally, more research is needed on cross-country differences in associations between diabetes and disabilities.

Although literature has frequently shown that diabetes has a role in the disablement of people, further research is needed to extend the current limited knowledge about this association [9]. The current study tested cross-country differences in association between diabetes and ADLs.

## Methods

Data came from Research on Early Life and Aging Trends and Effects (RELATE), that included seven different studies [10]. This analysis included 25,372 individuals who were age 40 or older. These individuals were sampled in the following seven countries: China, Mexico, Barbados, Brazil, Chile, Cuba, and Uruguay. All surveys were fully in compliance with the Helsinki Declaration on ethical principles for medical research involving humans. Different institutional review boards approved participating surveys.

The following countries were participating in RELATE project and represented countries from a diverse range in national income levels: Barbados represents high income countries; Cuba, Uruguay, Chile, Brazil, and Mexico represent upper middle income countries; and China represents lower middle income countries [11].

Although most country-specific surveys had sampling weights, as recommended, the current study did not apply sampling weights. The main reason was that sampling weights were not applicable to data from China (China Health and Nutrition Study; CHNS).

### Measures

#### ***Demographic characteristics***

The study measured age (continuous variable) and gender (dichotomous variable of male and female) as two demographic variables.

#### ***Socio-economic status***

The study measured education (four level categorical variable of 1) no schooling, 2) primary to elementary, 3) secondary to intermediate, and 4) higher), and income(continuous variable) as socio-economic status.

#### ***Obesity and exercise***

The study measured obesity and exercise based on self reported data. BMI larger than 30 was considered as obesity.

#### ***Main outcome***

Seven activities of daily living (ADLs) were measured by a modified Barthel index [12-15]. ADL items that were included in this study included bath, dress, toilet, transfer, heavy, shopping, and meals. These items have been frequently used to assess ADLs in the community sample [16,17].

### Data analysis

Data analysis was conducted using SPSS for Windows. We used country specific logistic regressions to determine if diabetes is associated with ADLs, and if this link is independent of socio-economic factors (education, and income), obesity and exercise. In all models, at least one ADL limitation was our outcome. In *Model I*, we only adjusted for age and gender. In *Model II*, we also adjusted for education and income. In *Model III*, we also adjusted for obesity and exercise.

## Results

Table 1 shows country differences by means of mean age, education, income, and ADLs. Mean age was lowest in China, and highest in Brazil. ADL limitation was highest in Chile, and lowest in Uruguay. Education was lowest in Brazil, and highest in Cuba. Income was highest in Uruguay and lowest in Cuba.

**Table 1:** Age, income, education, and activities of daily living in different countries among 25,372 community sample of adults

	**Min**	**Max**	**Mean**	**SD**
**China**				
Age	40	100	60.60	10.30
Income	0	500000	8645.24	15404.23
Education	1	4	2.18	0.85
Activities of daily living	0	7	0.63	1.35
**Mexico**				
Age	40	97	68.81	8.41
Income	0	714285	11716.48	29825.89
Education	1	4	2.02	0.75
Activities of daily living	0	7	0.71	1.23
**Barbados**				
Age	60	97	72.17	8.13
Income	0	804800	9321.03	37361.75
Education	1	4	2.22	0.56
Activities of daily living	0	7	0.32	0.84
**Brazil**				
Age	60	100	72.94	8.26
Income	0	99900	3705.36	7495.84
Education	1	4	1.83	0.68
Activities of daily living	0	7	0.84	1.36
**Chile**				
Age	60	99	71.44	8.00
Income	0	989232	279282.30	266840.20
Education	1	4	2.19	0.81
Activities of daily living	0	7	0.92	1.49
**Cuba**				
Age	60	102	71.63	8.66
Income	0	109992	1456.25	5071.77
Education	1	4	2.46	0.70
Activities of daily living	0	7	0.69	1.28
**Uruguay**				
Age	60	97	70.67	7.28
Income	0	960000	39607.53	71022.13
Education	1	4	2.37	0.78
Activities of daily living	0	7	0.46	0.95

### Model I

Based on the first model, with an exception of China, diabetes was associated with ADL, and this link was independent of age and gender. Gender and age were associated with ADL in all countries (Table 2).

**Table 2:** Results of models I, with age and gender as covariates among 25,372 community sample of adults

						**95% C.I. for EXP(B)**	
	**B**	**S.E.**	**Wald**	**Sig.**	**Exp(B)**	**Lower**	**Upper**
**China**							
Diabetes	.046	.057	0.636	.425	1.047	0.936	1.171
Age	.119	.002	4322.269	< .001	1.126	1.122	1.13
Female gender	.823	.038	474.844	< .001	2.277	2.115	2.452
**Mexico**							
Diabetes	.294	.149	3.866	.049	1.342	1.001	1.798
Age	.073	.008	78.043	< .001	1.075	1.058	1.093
Female gender	1.087	.134	66.085	< .001	2.966	2.282	3.855
**Barbados**							
Diabetes	.575	.151	14.59	< .001	1.778	1.323	2.389
Age	.064	.008	65.319	< .001	1.066	1.05	1.083
Female gender	.625	.143	19.177	< .001	1.869	1.413	2.472
**Brazil**							
Diabetes	.298	.121	6.036	.014	1.347	1.062	1.708
Age	.075	.006	164.576	< .001	1.078	1.066	1.09
Female gender	.973	.098	98.472	< .001	2.646	2.183	3.207
**Chile**							
Diabetes	.372	.176	4.494	.034	1.451	1.029	2.048
Age	.065	.008	69.835	< .001	1.067	1.051	1.083
Female gender	1.095	.134	66.678	< .001	2.99	2.299	3.89
**Cuba**							
Diabetes	.378	.138	7.532	.006	1.459	1.114	1.91
Age	.062	.006	112.843	< .001	1.064	1.052	1.076
Female gender	1.009	.112	80.636	< .001	2.742	2.2	3.417
**Uruguay**							
Diabetes	.323	.172	3.532	.060	1.382	0.986	1.936
Age	.06	.008	54.291	< .001	1.062	1.045	1.079
Female gender	.785	.133	34.711	< .001	2.193	1.689	2.847

### Model II

Based on Model II that also controlled for education and income, education explained the association between diabetes and limitation in ADL in Mexico and Uruguay. Based on this model, gender and age were associated with ADL in all countries. However, education was not correlated with ADL in Barbados, Cuba, and Uruguay. Income was linked to ADL in China and Brazil (Table 3).

**Table 3:** Results of models II, with age, gender and socio-economic status as covariates among 25,372 community sample of adults

						**95% C.I. for EXP(B)**	
	**B**	**S.E.**	**Wald**	**Sig.**	**Exp(B)**	**Lower**	**Upper**
**China**							
Diabetes	.062	.059	1.081	.299	1.063	.947	1.194
Age	.118	.002	4050.944	.000	1.125	1.121	1.129
Female gender	.730	.042	305.224	< .001	2.075	1.912	2.252
Education	-.169	.026	41.884	< .001	.844	.802	.889
Income	.000	.000	4.355	.037	1.000	1.000	1.000
**Mexico**							
Diabetes	.236	.151	2.438	.118	1.266	.942	1.701
Age	.070	.008	71.441	< .001	1.073	1.056	1.091
Female gender	1.045	.135	60.226	< .001	2.843	2.184	3.701
Education	-.250	.078	10.385	.001	.779	.669	.907
Income	.000	.000	.269	.604	1.000	1.000	1.000
**Barbados**							
Diabetes	.666	.163	16.737	< .001	1.947	1.415	2.680
Age	.064	.009	52.773	< .001	1.066	1.047	1.084
Female gender	.588	.159	13.715	< .001	1.800	1.319	2.457
Education	-.137	.122	1.259	.262	.872	.687	1.107
Income	.000	.000	1.540	.215	1.000	1.000	1.000
**Brazil**							
Diabetes	.290	.123	5.562	.018	1.336	1.050	1.701
Age	.070	.006	135.643	< .001	1.072	1.060	1.085
Female gender	.923	.099	86.452	< .001	2.516	2.071	3.056
Education	-.237	.069	11.764	< .001	.789	.689	.903
Income	.000	.000	18.748	< .001	1.000	1.000	1.000
**Chile**							
Diabetes	.392	.180	4.750	.029	1.480	1.040	2.105
Age	.065	.008	67.121	< .001	1.067	1.051	1.084
Female gender	1.070	.137	60.798	< .001	2.917	2.229	3.817
Education	-.248	.062	16.089	< .001	.781	.692	.881
Income	.000	.000	1.143	.285	1.000	1.000	1.000
**Cuba**							
Diabetes	.381	.138	7.578	.006	1.463	1.116	1.919
Age	.058	.006	93.065	< .001	1.059	1.047	1.072
Female gender	.980	.113	75.287	< .001	2.666	2.136	3.327
Education	-.222	.073	9.251	.002	.801	.694	.924
Income	.000	.000	3.320	.068	1.000	1.000	1.000
**Uruguay**							
Diabetes	.227	.183	1.541	.214	1.255	.877	1.798
Age	.052	.009	36.573	< .001	1.054	1.036	1.072
Female gender	.820	.142	33.140	< .001	2.270	1.717	3.000
Education	-.238	.074	10.441	.001	.788	.682	.911
Income	.000	.000	.178	.673	1.000	1.000	1.000

### Model III

Based on Model III that also controlled for obesity and exercise, in Cuba and Brazil, exercise explained the link between diabetes and limitation in performing ADLs. Based on this model, the link between diabetes and ADL was independent of our covariates only in Chile and Barbados. Based on this model, gender and age were associated with ADL in all countries. Obesity was linked to ADL only in Barbados. Exercise was linked to ADL in all countries (Table 4).

**Table 4:** Results of models III, with age, gender, socio-economic status, exercise and obesity as covariates among 25,372 community sample of adults

						**95% C.I. for EXP(B)**	
	**B**	**S.E.**	**Wald**	**Sig.**	**Exp(B)**	**Lower**	**Upper**
**China**							
Diabetes	.036	.316	.013	.909	1.037	.558	1.926
Age	.103	.009	134.296	< .001	1.108	1.089	1.128
Female gender	.836	.136	37.870	< .001	2.307	1.768	3.011
Education	-.128	.054	5.520	.019	.880	.791	.979
Income	.000	.000	3.445	.063	1.000	1.000	1.000
Exercise	-.711	.160	19.680	< .001	.491	.359	.673
Obesity	-.390	.326	1.427	.232	.677	.357	1.284
**Mexico**							
Diabetes	.185	.166	1.236	.266	1.203	.868	1.668
Age	.062	.009	42.516	< .001	1.064	1.044	1.084
Female gender	1.042	.153	46.221	< .001	2.836	2.100	3.830
Education	-.238	.089	7.106	.008	.788	.661	.939
Income	.000	.000	.003	.953	1.000	1.000	1.000
Exercise	-.569	.160	12.663	< .001	.566	.413	.774
Obesity	.079	.154	.260	.610	1.082	.800	1.463
**Barbados**							
Diabetes	.438	.178	6.072	.014	1.550	1.094	2.196
Age	.053	.010	27.477	< .001	1.054	1.034	1.075
Female gender	.478	.176	7.411	.006	1.613	1.143	2.275
Education	-.038	.131	.084	.772	.963	.744	1.245
Income	.000	.000	2.459	.117	1.000	1.000	1.000
Exercise	-.602	.176	11.730	.001	.548	.388	.773
Obesity	.513	.185	7.716	.005	1.671	1.163	2.400
**Brazil**							
Diabetes	.125	.137	.831	.362	1.133	.866	1.484
Age	.061	.007	78.208	< .001	1.063	1.048	1.077
Female gender	.974	.114	72.657	< .001	2.649	2.117	3.314
Education	-.210	.081	6.757	.009	.811	.692	.950
Income	.000	.000	9.882	.002	1.000	1.000	1.000
Exercise	-1.075	.143	56.711	< .001	.341	.258	.451
Obesity	.198	.132	2.235	.135	1.219	.940	1.580
**Chile**							
Diabetes	.413	.188	4.848	.028	1.512	1.046	2.183
Age	.061	.008	52.554	< .001	1.063	1.045	1.080
Female gender	1.068	.145	54.130	< .001	2.911	2.190	3.870
Education	-.213	.064	11.033	.001	.808	.713	.916
Income	.000	.000	.587	.443	1.000	1.000	1.000
Exercise	-.764	.168	20.648	< .001	.466	.335	.648
Obesity	.012	.141	.007	.933	1.012	.767	1.335
**Cuba**							
Diabetes	.278	.149	3.472	.062	1.320	.986	1.768
Age	.047	.007	50.995	< .001	1.048	1.035	1.061
Female gender	.956	.124	59.358	< .001	2.601	2.040	3.318
Education	-.132	.080	2.736	.098	.876	.749	1.025
Income	.000	.000	1.888	.169	1.000	1.000	1.000
Exercise	-.729	.149	23.980	< .001	.483	.361	.646
Obesity	.263	.154	2.907	.088	1.301	.961	1.760
**Uruguay**							
Diabetes	.155	.197	.623	.430	1.168	.794	1.718
Age	.047	.009	25.884	< .001	1.049	1.030	1.068
Female gender	.724	.156	21.482	< .001	2.062	1.518	2.801
Education	-.196	.081	5.878	.015	.822	.701	.963
Income	.000	.000	.382	.536	1.000	1.000	1.000
Exercise	-1.413	.282	25.015	< .001	.244	.140	.424
Obesity	.112	.145	.594	.441	1.118	.842	1.485

## Discussion

Our study showed at least four important cross-country differences in the pattern of association between diabetes and disability; 1) in most (i.e. Mexico, Barbados, Brazil, Chile, Cuba, and Uruguay) but not all countries (China), diabetes seems to be associated with ADL, 2) in some countries (i.e. Mexico and Uruguay), education explains the association between diabetes and ADL limitation, 3) in some countries (i.e. Cuba and Brazil), exercise explains the link between diabetes and ADLs, and 4) in some countries (i.e. Chile and Barbados), the link between diabetes and ADL seems to be independent of our covariates/mediators.

Only in Chile and Barbados was the link between diabetes and ADL independent of all study covariates/mediators. Based on a study in Japan, history of diabetes, bone fractures, and heart diseases contributed to some specific aspects of ADL disabilities, however, cerebrovascular disease influenced all aspects of ADL [18].

We showed that in Cuba and Brazil, physical activity might be a mechanism behind the link between diabetes and ADL. According to the literature, at least a part of the limitations in ADLs of patients with diabetes might be due to function in extremities. A study examined how hand disorders contribute to ADLs among elderly men with diabetes, and showed that limited joint motion (measured by prayer sign and Dupuytren's contracture) was more common in individuals with diabetes, compared to non-diabetics. Vibrotactile sense was impaired symmetrically in the index and little fingers in diabetics [19].

Peripheral artery disease and peripheral nerve dysfunction are known causes of diabetes -related disability [9,20], and may explain more than 30% of the association of diabetes with disability related dysfunction in physical activity. Physical activity is particularly important for people with diabetes, because being physically active can improve the body's ability to use insulin and facilitate weight loss [21-25]. In the United States, for example, only one-third of individuals with diabetes and obesity are physically active [26,27]. Interventions need to enhance physical activity of patients, and this may break the cycle by which diabetes causes limitation in performing ADLs.

The current study failed to show mediating or confounding effects of obesity on the association between diabetes and ADLs. However, obesity had an independent association with ADLs in one of the seven countries (Barbados). Literature suggests that obesity and overweight may mediate or confound the link between diabetes and disability. In a survey among obese diabetic individuals, ADL was linked to current exercise, using exercise programs, and self-reported weight history [28]. In a qualitative study among obese (body mass index [BMI] > 30 kg/m2) individuals with diabetes, patients believed that their performance of daily activities would improve with weight loss of 5–10% of body weight [29].

The ability to remain physically active is an essential aspect of quality of life and is critical for the preservation of independence among patients with diabetes. Lower extremity function is a strong predictor of poor health outcomes, including disability, hospitalization, and death among patients [30,31]. Recently, a search for potentially modifiable conditions associated with impaired mobility and lower extremity function identified several socio-demographic and behavioral characteristics, along with acute and chronic medical conditions [32,33]. Among the latter group, diabetes has been shown to be consistently a correlate of poor extremity performance [34,35].

Several impairments and comorbidities are involved in the disablement process associated with diabetes. Obesity, visual impairment, and cardiovascular diseases may mediate the association between diabetes and disability among diabetic patients. These conditions are important causes of different aspects of physical dysfunction in older individuals [36,37].

The association between diabetes and ADLs did not stay significant in the multivariable models in most countries. The literature is also not informative about the question of whether or not the link between diabetes and disability is independent of other factors that contribute to disability. We know that several conditions that may contribute to the impairment in ADLs are more prevalent in diabetic patients than controls. These conditions may be the actual mechanisms by which diabetes is associated with physical disability. Some of these conditions include cardiovascular diseases, peripheral neuropathy, obesity, and visual deficits [35].

Our findings are important for different reasons. As very few studies have shown exact mechanisms by which disability is seen among patients with diabetes [35], our knowledge about the mechanisms underlying the disability among diabetic patients is limited. Further research is needed to know which complication of the disease plays a more important role in the disablement process. Further research is also needed on possible synergistic effects of different conditions associated with diabetes [38]. In addition, prevalence of diabetes is expected to increase considerably in the next decades [38]. And knowledge from similar studies may be important for planning strategies aimed at preventing or slowing functional decline in older persons and for tertiary prevention in subjects with diabetes [9].

Our study has strengths and limitations. This was a cross-national survey composed of different surveys conducted in seven countries [10]. Research on Early Life and Aging Trends and Effects (RELATE) did not collect data on duration of diabetes, or mental health of the participants. Duration of diabetes is known to be strongly linked to disability among patients with diabetes. We also did not collect data on degree of metabolic imbalance, as we know loss of control of glucose may be a mediator for the disabling effect of diabetes [9]. Based on the literature, high prevalence of depression among diabetic patients may explain a substantial proportion of the excess risk of disability associated with diabetes. Depression is common among patients with diabetes [39-41]. Longitudinal studies are warranted to elucidate the role of depression in the pathway from diabetes to disability. Our study is one of very few studies that contribute to our understandings of cross – country differences in morbidity related to diabetes. To study cross – country differences in mental health associates of diabetes, one study used data from World Mental Health, and showed that with a consistent pattern, in all countries, mood and anxiety disorders occurred with somewhat greater frequency among persons with diabetes than those without diabetes, and the strength of association did not differ significantly across countries [42]. It is essential to conduct more research to test if the association between diabetes and disability is independent of other chronic conditions or not [9].

Our study sheds light on cross – country differences in factors associated with well-being. Considerable differences in morbidity, life satisfaction, and well-being have been shown across the globe [43,44]. The World Values Survey, European Values Study, Eurobarometer, and Latinobarometer, have consistently shown that life expectancy, physical health, all-cause mortality, and also subjective well-being vary across countries [45-48].

## Conclusion

To conclude, our study showed considerable differences in the association between diabetes and disability across countries. The study also suggests that there might be some cross-country differences in the factors that may explain this link.

## Competing interests

The authors declare that they have no competing interests.

## Authors’ contributions

SA designed the study and conducted data analysis, MML and RML conducted the literature review and drafted the manuscript. All authors read and approved the final manuscript.

## References

[B1] Danaei G Finucane MM Lu Y Singh GM Cowan MJ Paciorek CJ National, regional, and global trends in fasting plasma glucose and diabetes prevalence since 1980: systematic analysis of health examination surveys and epidemiological studies with 370 country-years and 2.7 million participants Lancet 2011 378 31 40 10.1016/S0140-6736(11)60679-X 21705069

[B2] International Diabetes Foundation Global Burdenhttp://www.idf.org/diabetesatlas/5e/the-global-burden

[B3] Ryerson B Tierney EF Thompson TJ Engelgau MM Wang J Gregg EW Geiss LS Excess physical limitations among adults with diabetes in the US population, 1997–1999 Diabetes Care 2003 26 206 10 10.2337/diacare.26.1.206 12502682

[B4] Gregg EW Beckles GL Williamson DF Leveille SG Langlois JA Engelgau MM Narayan KM Diabetes and physical disability among older US adults Diabetes Care 2000 23 1272 7 10.2337/diacare.23.9.1272 10977018

[B5] Volpato S Blaum C Resnick H Ferrucci L Fried LP Guralnick JM Women’s Health and Aging Study Diabetes Care 2002 25 678 83 10.2337/diacare.25.4.678 11919124

[B6] Von Korff M Katon W Lin EH Simon G Ludman E Oliver M Ciechanowski P Rutter C Bush T Potentially modifiable factors associated with disability among people with diabetes Psychosom Med 2005 67 2 233 40 10.1097/01.psy.0000155662.82621.50 15784788

[B7] Bourdel-Marchasson I Dubroca B Manciet G Decamps A Emeriau JP Dartigues JF Prevalence of diabetes and effect on quality of life in older French living in the community: the PAQUID Epidemiological Survey J Am Geriatr Soc 1997 45 295 301 9063274 10.1111/j.1532-5415.1997.tb00943.x

[B8] Ferrucci L Penninx BW Leveille SG Corti MC Pahor M Wallace R Harris TB Havlik RJ Guralnik JM Characteristics of nondisabled older persons who perform poorly in objective tests of lower extremity function J Am Geriatr Soc 2000 48 1102 1110 10983911 10.1111/j.1532-5415.2000.tb04787.x

[B9] Volpato S Blaum C Resnick H Ferrucci L Fried LP Guralnik JM Women’s Health and Aging Study. Comorbidities and impairments explaining the association between diabetes and lower extremity disability: the Women's Health and Aging Study Diabetes Care 2002 25 4 678 83 10.2337/diacare.25.4.678 11919124

[B10] McEniry M Research on Early Life and Aging Trends and Effects (RELATE): A Cross-National Study. ICPSR34241-v1 2013 Ann Arbor, MI: Inter-university Consortium for Political and Social Research [distributor] 06 12 10.3886/ICPSR34241.v1 Persistent URL: http://dx.doi.org/10.3886/ICPSR34241.v1

[B11] The World Bank How we Classify Countrieshttp://data.worldbank.org/about/country-classifications

[B12] Mahoney FI Barthel D Functional evaluation: the Barthel Index Md State Med J 1965 14 56 61 14258950

[B13] Loewen SC Anderson BA Predictors of stroke outcome using objective measurement scales Stroke 1990 21 78 81 10.1161/01.STR.21.1.78 2300994

[B14] Gresham GE Phillips TF Labi ML ADL status in stroke: relative merits of three standard indexes Arch Phys Med Rehabil 1980 61 355 358 7406673

[B15] Collin C Wade DT Davies S Horne V The Barthel ADL Index: a reliability study Int Disability Study 1988 10 61 63 10.3109/09638288809164103 3403500

[B16] Dudgeon BJ Hoffman JM Ciol MA Shumway-Cook A Yorkston KM Chan L Managing activity difficulties at home: a survey of Medicare beneficiaries Arch Phys Med Rehabil 2008 89 7 1256 61 10.1016/j.apmr.2007.11.038. Epub 2008 Jun 13 10.1016/j.apmr.2007.11.038 18534553 PMC3425800

[B17] Ostchega Y Harris TB Hirsch R Parsons VL Kington R The prevalence of functional limitations and disability in older persons in the US: data from the National Health and Nutrition Examination Survey III J Am Geriatr Soc 2000 48 9 1132 5 10983915 10.1111/j.1532-5415.2000.tb04791.x

[B18] Kishimoto M Ojima T Nakamura Y Yanagawa H Fujita Y Kasagi F Kodama K Ueda K Suzuki S Kagamimori S Relationship between the level of activities of daily living and chronic medical conditions among the elderly J Epidemiol 1998 8 5 272 7 10.2188/jea.8.272 9884476

[B19] Cederlund RI Thomsen N Thrainsdottir S Eriksson KF Sundkvist G Dahlin LB Hand disorders, hand function, and activities of daily living in elderly men with type 2 diabetes J Diabetes Complications 2009 23 1 32 9 10.1016/j.jdiacomp.2007.09.002 18413154

[B20] Dermott MM Fried L Simonsick E Ling S Guralnik JM Asymptomatic peripheral arterial disease is independently associated with impaired lower extremity functioning: the Women’s Health and Aging Study Circulation 2000 101 1007 1012 10.1161/01.CIR.101.9.1007 10704168

[B21] Colberg SR Sigal RJ Fernhall B Regensteiner JG Blissmer BJ Rubin RR Chasan-Taber L Albright AL Braun B American College of Sports Medicine; American Diabetes Association: exercise and type 2 diabetes: the American College of Sports Medicine and the American Diabetes Association: joint position statement Diabetes Care 2010 33 e147 e167 10.2337/dc10-9990 21115758 PMC2992225

[B22] De Feo P Di Loreto C Ranchelli A Fatone C Gambelunghe G Lucidi P Santeusanio F Exercise and diabetes Acta Biomed 2006 77 Suppl 1 14 17 16921608

[B23] Kirk A De Feo P Strategies to enhance compliance to physical activity for patients with insulin resistance Appl Physiol Nutr Metab 2007 32 549 556 10.1139/H07-023 17510696

[B24] Balducci S Zanuso S Nicolucci A De Feo P Cavallo S Cardelli P Fallucca S Alessi E Fallucca F Pugliese G Italian Diabetes Exercise Study (IDES) Investigators: effect of an intensive exercise intervention strategy on modifiable cardiovascular risk factors in subjects with type 2 diabetes mellitus Arch Intern Med 2010 170 1794 1803 10.1001/archinternmed.2010.380 21059972

[B25] National Institute of Diabetes and Digestive and Kidney Diseases What I Need to Know About Physical Activity and Diabetes 2008 http://www.diabetes.niddk.nih.gov (accessed February 2011)

[B26] Nelson KM Reiber G Boyko EJ Diet and exercise among adults with type 2 diabetes: findings from the Third National Health and Nutritional Examination Survey (NHANES III) Diabetes Care 2002 25 1722 1728 10.2337/diacare.25.10.1722 12351468

[B27] Moratto EH Hill JO Wyatt HR Ghushchyan V Sullivan PW Physical activity in U.S. adults with diabetes and at risk for developing diabetes Diabetes Care 2007 30 203 209 10.2337/dc06-1128 17259482

[B28] Hayes RP Nelson DR Meldahl ML Curtis BH Ability to perform daily physical activities in individuals with type 2 diabetes and moderate obesity: a preliminary validation of the Impact of Weight on Activities of Daily Living Questionnaire Diabetes Technol Ther 2011 13 7 705 12 10.1089/dia.2011.0027 21504333

[B29] Curtis B Hayes R Fehnel S Zografos L Assessing the effect of weight and weight loss in obese persons with type 2 diabetes Diabetes Metab Syndr Obes 2008 1 13 23 21437152 10.2147/dmso.s4237 PMC3052714

[B30] Guralnik JM Ferrucci L Simonsick EM Salive ME Wallace RB Lower-extremity function in persons over the age of 70 years as a predictor of subsequent disability N Engl J Med 1995 332 556 561 10.1056/NEJM199503023320902 7838189 PMC9828188

[B31] Penninx BW Ferrucci L Leveille SG Rantanen T Pahor M Guralnik JM Lower extremity performance in nondisabled older persons as a predictor of subsequent hospitalization J Gerontol A Biol Sci Med Sci 2000 55 M691 M697 10.1093/gerona/55.11.M691 11078100

[B32] Ensrud KE Nevitt MC Yunis C Cauley JA Seeley DG Fox KM Cummings SR Correlates of impaired function in older women J Am Geriatr Soc 1994 42 481 489 8176141 10.1111/j.1532-5415.1994.tb04968.x

[B33] Ferrucci L Guralnik JM Pahor M Corti MC Havlik RJ Hospital diagnoses, Medicare charges, and nursing home admissions in the year when older persons become severely disabled JAMA 1997 277 728 734 10.1001/jama.1997.03540330050034 9042845

[B34] Perkowski LC Stroup-Benham CA Markides KS Lichtenstein MJ Angel RJ Guralnik JM Goodwin JS Lower-extremity functioning in older Mexican Americans and its association with medical problems J Am Geriatr Soc 1998 46 411 418 9560061 10.1111/j.1532-5415.1998.tb02459.x

[B35] Menz HB Lord SR St George R Fitzpatrick RC Walking stability and sensorimotor function in older people with diabetic peripheral neuropathy Arch Phys Med Rehabil 2004 85 2 245 52 Feb 10.1016/j.apmr.2003.06.015 14966709

[B36] Guccione AA Felson DT Anderson JJ Anthony JM Zhang Y Wilson PW Kelly-Hayes M Wolf PA Kreger BE Kannel WB The effects of specific medical conditions on the functional limitations of elders in the Framingham Study Am J Public Health 1994 84 351 358 10.2105/AJPH.84.3.351 8129049 PMC1614827

[B37] Fried LP Bandeen-Roche K Kasper JD Guralnik JM Association of comorbidity with disability in older women: the Women’s Health and Aging Study J Clin Epidemiol 1999 52 27 37 10.1016/S0895-4356(98)00124-3 9973071

[B38] Mokdad AH Ford ES Bowman BA Nelson DE Engelgau MM Vinicor F Marks JS Diabetes trends in the U.S.: 1990–1998 Diabetes Care 2000 23 1278 1283 10.2337/diacare.23.9.1278 10977060

[B39] Lustman PJ Anderson RJ Freedland KE de Groot M Carney RM Clouse RE Depression and poor glycemic control: a meta-analytic review of the literature Diabetes Care 2000 23 934 942 10.2337/diacare.23.7.934 10895843

[B40] Talbot F Nouwen A A review of the relationship between depression and diabetes in adults Diabetes Care 2000 23 1556 1562 10.2337/diacare.23.10.1556 11023152

[B41] Penninx BW Guralnik JM Ferrucci L Simonsick EM Deeg DJ Wallace RB Depressive symptoms and physical decline in community-dwelling older persons JAMA 1998 279 1720 1726 10.1001/jama.279.21.1720 9624025

[B42] Lin EH Korff MV Alonso J Angermeyer MC Anthony J Bromet E Bruffaerts R Gasquet I de Girolamo G Gureje O Haro JM Karam E Lara C Lee S Levinson D Ormel JH Posada-Villa J Scott K Watanabe M Williams D Mental disorders among persons with diabetes–results from the World Mental Health Surveys J Psychosom Res 2008 65 6 571 80 10.1016/j.jpsychores.2008.06.007 19027447 PMC3672403

[B43] Fabrega JJ The study of disease in relation to culture Behav Sci 1972 17 183 203 10.1002/bs.3830170202 5011956

[B44] Gureje O Ustun TB Simon GE The syndrome of hypochondriasis: a cross-national study in primary care Psychol Med 1997 27 1001 10 10.1017/S0033291797005345 9300506

[B45] Easterlin RA McVey LA Switek M Sawangfa O Zweig JS The happiness-income paradox revisited Proc Natl Acad Sci U S A 2010 107 52 22463 8 10.1073/pnas.1015962107 21149705 PMC3012515

[B46] Jen MH Sund ER Johnston R Jones K Trustful societies, trustful individuals, and health: an analysis of self-rated health and social trust using the World Value Survey Health Place 2010 16 5 1022 9 10.1016/j.healthplace.2010.06.008 20621543

[B47] Jen MH Jones K Johnston R Global variations in health: evaluating Wilkinson's income inequality hypothesis using the World Values Survey Soc Sci Med 2009 68 4 643 53 10.1016/j.socscimed.2008.11.026 19095338

[B48] Kim D Kawachi I Hoorn SV Ezzati M Is inequality at the heart of it? Cross-country associations of income inequality with cardiovascular diseases and risk factors Soc Sci Med 2008 66 8 1719 32 10.1016/j.socscimed.2007.12.030 18280021

