# Correction: Cross-country differences in the association between diabetes and disability

**DOI:** 10.1186/2251-6581-13-73

**Published:** 2014-07-10

**Authors:** Shervin Assari, Reza Moghani Lankarani, Maryam Moghani Lankarani

**Affiliations:** 1 Department of Health Behavior and Health Education, University of Michigan School of Public Health, 1415 Washington Heights, Ann Arbor, MI 48109-2029, USA; 2 Center for Research on Ethnicity, Culture and Health, School of Public Health, University of Michigan, 1415 Washington Heights, Ann Arbor, MI 48109-2029, USA; 3 Tehran University of Medical Sciences, Tehran, Iran; 4 Medicine and Health Promotion Institute, Tehran, Iran; 5 Universal Network for Health Information Dissemination and Exchange (UNHIDE), Tehran, Iran

## Correction

The acknowledgment section of a recent manuscript published by Assari et al., [1] should be corrected as below:

Publication of this manuscript was possible with the Cornely Fellowship fund awarded by the Center for Research on Ethnicity, Culture and Health, School of Public Health, University of Michigan to the first author, Shervin Assari.
